# Big data analysis of endovascular treatment of intracranial aneurysms: a bibliometric analysis of the top 100 most cited articles

**DOI:** 10.1055/s-0042-1758650

**Published:** 2022-12-29

**Authors:** Shuai Zhang, Dong Liu, Jiao Wang, Ming Lv

**Affiliations:** 1 Beijing Jingmei Group General Hospital, Department of Neurosurgery, Beijing, China.; 2 Capital Medical University, Beijing Tian Tan Hospital, Department of Neurology, Beijing, China.; 3 Beijing Jingmei Group General Hospital, Department of Emergency, Beijing, China.; 4 Capital Medical University, Beijing Tiantan Hospital, Beijing Neurosurgical Institute, Department of Interventional Neuroradiology, Beijing, China.

**Keywords:** Intracranial Aneurysm, Subarachnoid Hemorrhage, Endovascular Procedures, Bibliometrics, Aneurisma Intracraniano, Hemorragia Subaracnóidea, Procedimentos Endovasculares, Bibliometria

## Abstract

**Background** The impact of a literature report on a particular subject can be measured by its number of citations.

**Objective** The purpose of this study was to evaluate the characteristics of the literature reports in the field of endovascular treatment of intracranial aneurysms (IAs) by analyzing the top 100 most cited articles. It should be noted that the focus of this study is to describe the bibliometric characteristics.

**Methods** This bibliometric analysis dedicated only to endovascular treatment of IAs in our study. We searched the top 100 most cited articles in the field of endovascular treatment of IAs using the search tool of the Web of Science (WOS). We evaluated the characteristics of these high-impact publications, including publication year, category, journal, author's country, etc.

**Results** The top 100 most cited articles were cited 281.3 times, on average. The United States has published the most articles every year compared with other countries. These highly cited articles are mostly published in the Journal of Neurosurgery. Eighty-six of the top 100 most cited articles were clinical studies.

**Conclusion** The bibliometric analysis provides insight over the development and the growing trend in endovascular treatment of IAs. This study can help researchers better understand the global overview of this field, and it also provides leads about promising areas of future research and potential collaborations.

## Resumo

**Antecedentes** O impacto de um relato de literatura sobre um determinado assunto pode ser medido pelo seu número de citações.

**Objetivo** O objetivo deste estudo foi avaliar as características dos relatos da literatura na área de tratamento endovascular de aneurismas intracranianos (AIs), analisando os 100 artigos mais citados. Ressalta-se que o foco deste estudo é descrever as características bibliométricas.

**Métodos** Esta análise bibliométrica foi dedicada apenas ao tratamento endovascular dos AIs em nosso estudo. Pesquisamos os 100 artigos mais citados na área de tratamento endovascular de AIs usando a ferramenta de busca da *Web of Science* (WOS). Avaliamos as características dessas publicações de alto impacto, incluindo ano de publicação, categoria, periódico, país do autor etc.

**Resultados** Os 100 artigos mais citados foram citados 281,3 vezes, em média. Os Estados Unidos publicaram o maior número de artigos todos os anos em comparação com outros países. Esses artigos altamente citados são publicados principalmente no *Journal of Neurosurgery* . Oitenta e seis dos 100 artigos mais citados eram estudos clínicos.

**Conclusão** A análise bibliométrica fornece informações sobre o desenvolvimento e a tendência crescente no tratamento endovascular das AI. Este estudo pode ajudar os pesquisadores a entender melhor a visão global desse campo e também fornece pistas sobre áreas promissoras de pesquisas futuras e potenciais colaborações.

## INTRODUCTION

Endovascular technology has been used to embolize intracranial aneurysms (IAs) since the Guglielmi detachable coil (GDC) was introduced for clinical use in 1990. 1 Currently, an increasing number of patients with ruptured or unruptured IAs are being treated with endovascular therapy. The endovascular treatment methods for IAs include simple coil embolization, stent-assisted embolization, and placement of Flow Diversion Device. According to the international multi-center randomized controlled study (International Subarachnoid Aneurysm Trial, ISAT), endovascular treatment was better than neurosurgical clipping and reduced the mortality and disability rate of IAs patients. Moreover, the complication rate of endovascular treatment was lower than that of craniotomy. But the rebleeding rate was higher than craniotomy. 2 3 To our knowledge, endovascular therapy is more suitable for patients with advanced age, weak physique, or severe organic diseases who cannot tolerate craniotomy, as well as for patients with posterior circulation, wide neck, or huge IAs.

Many researchers have focused on research reports on the endovascular treatment of IAs. We have learned that bibliometric analysis is an analytical method that provides comprehensive knowledge about a particular area of study and identifies current research hotspots or research directions. We aim to provide a new perspective through bibliometric analysis to understand the overall research status of endovascular treatment of IAs.

The impact of a research report can be evaluated by the number of citations. An overview of the research area can be obtained through bibliometric analysis of the most cited literature. There have been many reports of bibliometric analysis in many fields. However, the bibliometric analysis dedicated to endovascular treatment for IAs has not been reported. Therefore, the purpose of this study is to understand the development in the field of endovascular treatment of IAs and to use bibliometric methods to analyze the characteristics of the 100 most cited articles in the field.

## METHODS

This study was a bibliometric analysis, so ethical approval was not required.

### Search strategy

**Figure 1:**
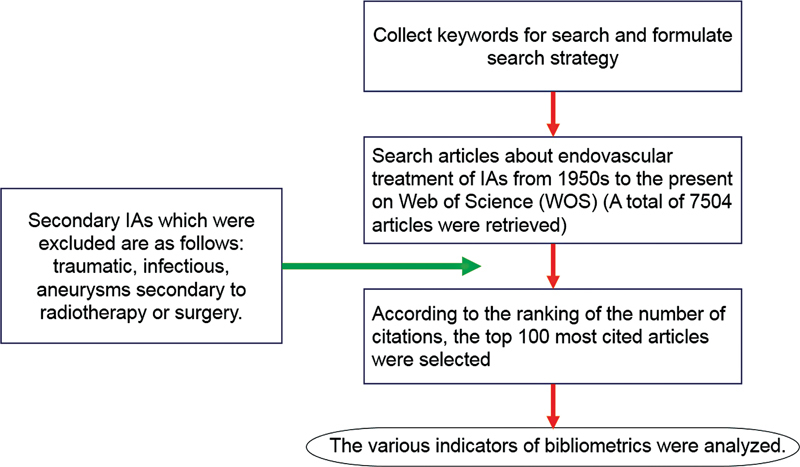
The flow chart of this study.

Web of Science (WOS) is an online database platform. It can provide information on the annual/total number of citations, authors, publication year, source journals, article types, citation indexes, etc. We searched the literature on endovascular treatment of IAs from the 1950s to the present day on WOS ( Figure 1 ).

In this study, the following secondary IAs were excluded: traumatic, infectious, and aneurysms secondary to radiotherapy or surgery. The articles were ranked based on the number of citations. The top 100 most cited articles were selected for analysis, and the full texts of these 100 articles were downloaded for analysis.

### Information collection and analysis

**Figure 2:**
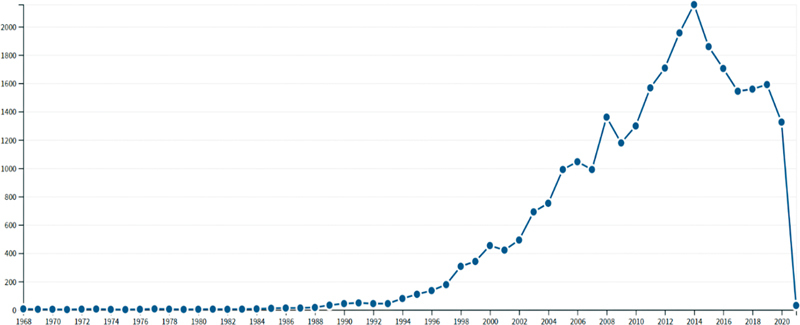
The annual citation number of the top 100 most cited articles.

We analyzed the top 100 most cited articles, and the following information of each article was obtained ( Figures 2 3 4 5 , Table 1 2 3 4 ): (1) the number of articles published each year; (2) the number of articles published in each country over the years; (3) the cooperative network relationship between countries; (4) the number of citations per year; (5) the details of the top 100 most cited articles (publication year, journal, first author, number of citations/number of citations per year); (5) the details of journals (number of citations, number of articles, average number of citations per article); (6) country of the main author; (7) article category (clinical research, review, basic research, guideline, commentary, expert consensus, protocol); (8) productive authors (number of articles, position on author list); (9) productive institutions (number of articles, citations).

**Figure 3:**
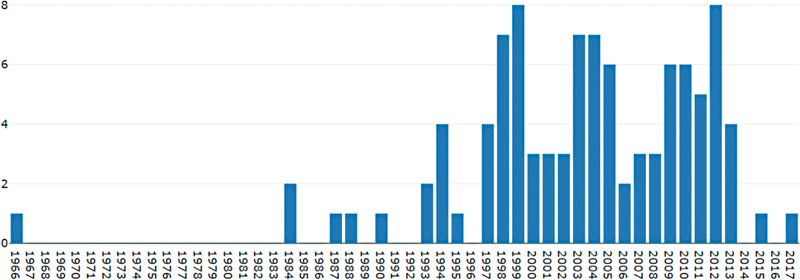
Number of articles per year for the top 100 most cited articles.

**Figure 4:**
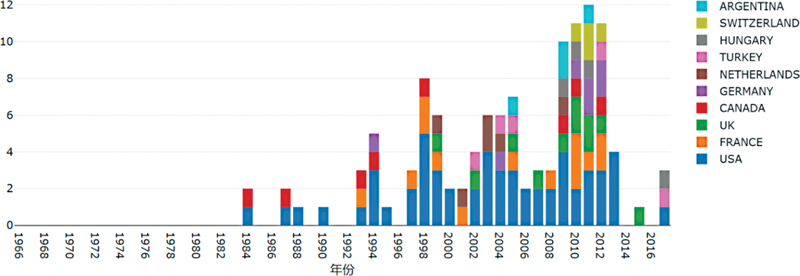
Annual number of the top 100 most cited articles in some countries (countries with more reports).

**Figure 5:**
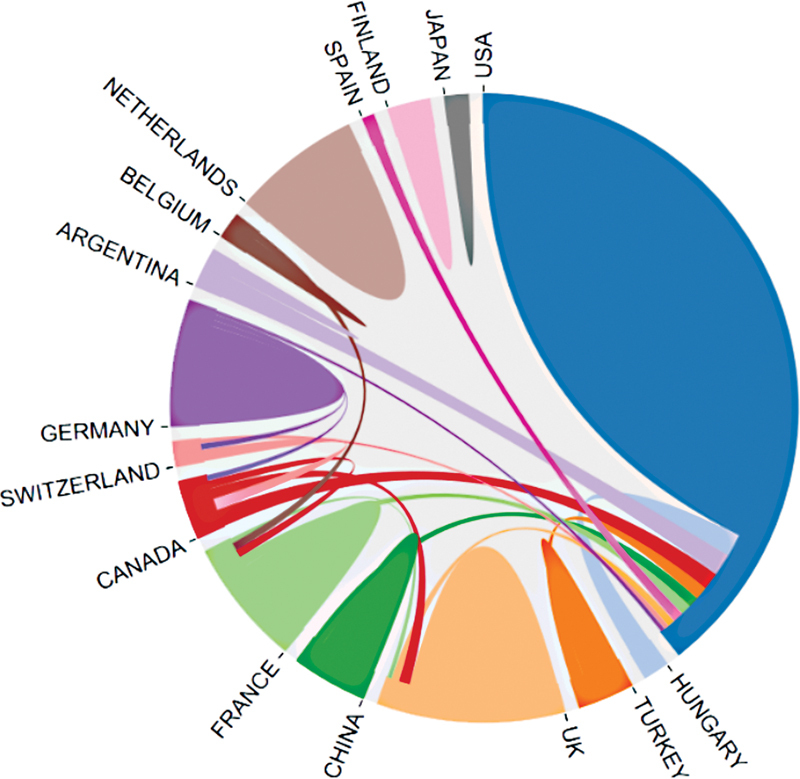
The schematic diagram of cooperation network between countries.

**Table 1:** Journals publishing the top 100 most cited articles on endovascular treatment of intracranial aneurysms

Journal	Citations (n)	Number of articles	Annual number of citations	Impact factor
Journal of Neurosurgery	150	24	6.3	3.96
Stroke	69	20	3.5	7.19
American Journal of Neuroradiology	68	15	4.5	3.38
Neurosurgery	90	15	6.0	4.85
Radiology	39	8	4.9	7.93
Lancet	40	5	8.0	60.39
Acta Neurochirurgica	6	2	3.0	1.81
Neuroradiology	7	2	3.5	2.24
Annals of Neurology	3	1	3.0	9.04
IEEE Transactions on Medical Imaging	1	1	1.0	6.68
Journal of NeuroInterventional Surgery	2	1	2.0	4.46
Journal of Neuroradiology	10	1	10.0	2.42
Lancet Neurology	4	1	4.0	30.03
Neurological Research	0	1	0	2.40
Neurology	5	1	5.0	8.77
Plos One	9	1	9.0	2.74
Surgical Neurology	6	1	6.0	0

**Table 2:** Publication area distribution of the top 100 most cited articles on endovascular treatment of IAs

Country	Number of articles
USA	57
France	14
England	9
Canada	8
Germany	7
Netherlands	6
Turkey	5
Argentina	4
Hungary	4
Scotland	4
Switzerland	4
Finland	3
Japan	3
China	2
Australia	1
Belgium	1
Ireland	1
Spain	1
USSR	1

**Table 3:** Institutions that contributed 5 or more of the top 100 most cited articles

Institution	Number of articles	Citations (n)
University of California system	15	50
State University of New York (SUNY) system	10	48
New York University	8	87
State University of New York (SUNY) Buffalo	8	48
University of California Los Angeles	8	27
Barrow Neurological Institute	7	78
University of California San Francisco	7	27
David Geffen School of Medicine at UCLA	6	27
Elisabeth-TweeSteden Ziekenhuis (ETZ)	6	21
Mayo clinic	6	22
University of Oxford	6	60
Université de Paris	5	1

**Table 4:** Authors who contributed 5 or more of the 100 most cited articles

Author	Number of articles	Position on author list (number of articles)
Fiorella D	9	FA (4); FourA (1); SixA (1); SevA (1); TenA (1); 15thA (1)
Vinuela F	9	FA (1); SA (2); TA (2); Four A (1); SixA (1); EA (1)
Lanzino G	8	FA (1); SA (3); TA (2); FourA (1); TenA (1)
Duckwiler GR	8	SA (1); TA (3); FourA (3); 27thA(1)
Molyneux AJ	8	FA (4); TA (2); FifA (1); 55thA (1)
Nelson PK	7	FA (1); FourA (2); SevA (1); EA (1); NA (1); 22th A (1)
Albuquerque FC	6	SA (2); TA (2); FifA (1); 14th A (1)
Goblin YP	6	TA (1); FourA (3); FifA (1); SixA (1)
Hopkins LN	6	FourA (1); FifA (1); SixA (1); SevA (1); EA (1); 15thA (1)
Van Rooij WJ	6	SA (2); TA (3); FourA (1)
Guglielmi G	5	SA (2); TA (1); FifA (2)
Higashida RT	5	FA (2); SA (1); FourA (1); FifA (1)
kallmes DF	5	SA (1); FourA (2); FifA (1); SixA (1)
Mcdougall CG	5	FourA (1); SixA (2); EA (1); 14thA (1)
Sluzewski M	5	FA (4); SevA (1)
Wakhloo AK	5	FA (2); SA (1); TA (1); SevA (1)

Abbreviations: EA, eighth author; FA, first Author; FifA, fifth author; FourA, fourth author; NA, ninth author; SA, second author; SevA, seventh author; SixA, sixth author; TA, third author; TenA, tenth author.

The extraction of the above-mentioned information was completed by two reviewers (neurointerventional physicians). If there was a dispute, the task was handed over to the third reviewer (senior neurointerventional physician), who made the final decision.

## RESULTS

### The overall situation

A total of 7,504 articles were retrieved. The top 100 most cited articles that met the inclusion criteria for analysis were selected. The details of the top 100 most cited articles are shown in Supplementary Table S1 . The top 100 most cited articles were cited 281.3 times on average (range, 135–2254).

### The most cited articles on endovascular treatment of IAs

The most cited article was the manuscript titled “International Subarachnoid Aneurysm Trial (ISAT) of neurosurgical clipping versus endovascular coiling in 2,143 patients with ruptured intracranial aneurysms: a randomized trial,” which was published in Lancet in 2002. It has been cited 2,254 times, with an average of 112.7 citations per year.

### The annual citations number

As shown in Figure 2 , the annual number of citations of these articles increased in the beginning and then decreased over time. These articles were cited with the most in 2012.

### Time of publication, publication area distribution, annual number in some countries, and cooperation between countries

As shown in Figure 3 , these top 100 most cited articles were mainly published during 1998–2013. The number of articles published in the top contributing countries are shown in Figure 4 . It was not difficult to see that, in the field of endovascular treatment of IAs, the United States produced the most articles every year.

The countries with the top 100 most cited articles are listed in Table 3 . The United States has published the most articles (57), with a significantly higher number of articles than France. The United States cooperated the most with other countries as shown in the schematic diagram of cooperation network ( Figure 5 ).

### Journals publishing the top 100 most cited articles

The journals that have published the most number of articles are shown in Table 1 . Among them, the Journal of Neurosurgery has published the most articles (24), with a total of 150 citations. It was followed by the Journal of Stroke, which published 20 articles, with a total of 69 citations.

### High-yield countries/individuals

The institutions with more output (≥ 5 articles) are shown in Table 3 . The University of California system has the most output, with a total of 15 articles published. Studies from the University of California system have been cited 50 times. The authors with more output (≥ 5) are shown in Table 4 . Fiorella et al. have published four highly cited articles.

### Article category

Among the top 100 most cited articles, most (86) of them were clinical studies, only 8 were review studies, 3 were basic studies, and 2 were commentary articles.

## DISCUSSION

The rupture of IAs can cause subarachnoid hemorrhage leading to disastrous consequences in patients. 4 With the advancement of interventional therapy technology, the endovascular treatment of IAs has become an important treatment method. 5

The purpose of this study was to review the most highly cited articles in the field of endovascular treatment of IAs, and to explore the characteristics of research studies in this field. To the best of our knowledge, the bibliometric analysis of intracranial aneurysmal subarachnoid hemorrhage has been reported. 6 However, the bibliometric analysis dedicated to the endovascular treatment of IAs has not been reported yet. The bibliometric analysis in this study is a contrived in-depth summary based on previous work. We reviewed the 100 most cited reports in the field of endovascular treatment of IAs as the basis for understanding this type of treatment. Next, we will discuss the results of the bibliometric analysis. It should be noted that the focus of this study is to describe the bibliometric characteristics.

### The characteristics of the citation number per year and the publication time of the top 100 most cited articles

The number of annual citations gradually increased from 1986 to 2013 ( Figure 2 ). The top 100 most cited articles were mainly published between 1997 and 2013. This was also a period with rapid development of endovascular treatment of IAs. During this period, the technology of interventional materials developed rapidly. Practitioners in this field also communicated with each other more conveniently with the development of the internet and social media. These reasons may have led to higher production of the top 100 most-cited articles during that period.

After 2013, the number decreased gradually. This was consistent with the general literature being cited. These reports attracted more and more physicians' interest in the early stage as the technique became more popular. Gradually, the endovascular treatment for IAs became well known to many physicians and less attention was paid to this field. In addition, with the emergence of new treatment equipment and technologies, people's attention to traditional endovascular treatment technologies gradually decreased.

### Journals that published more articles

As shown in Table 1 , the top 100 most cited documents were mainly published in the publications Journal of Neurosurgery, Stroke, American Journal of Neuroradiology, etc. Among them, the Journal of Neurosurgery is a classic journal that publishes clinical and basic research on neurosurgery. It has published 24 of the top 100 most cited reports on endovascular treatment of IAs. Most of the highly cited reports were published in professional journals, which may be due to the fact that readers of comprehensive journals lack sufficient interest in reports on intravascular therapy of IAs compared with the readers of professional journals.

### Main contributing countries and the cooperation network between these countries

As shown in Table 2 , the United States published the highest number of papers among the top 100 ones, and that number was significantly higher than those of other countries. Developing countries have published fewer articles of the top 100 most cited articles. As shown in Figure 5 , the United States was the hub of the collaboration network, which is similar to other bibliometric analysis results.

The United States is the largest economy in the world and provides economic support to medical research worldwide. There are also many scientific and technological innovations in the United States that may accelerate the production of highly cited documents in this field. Other countries also seek to cooperate with the United States to promote their own development in this field.

It should be noted that although the United States has contributed the most in number, this does not mean that it has made the greatest contribution to this field. For example, the International Subarachnoid Aneurysm Trial (ISAT) is a multi-center study, and its research branch did not include the United States.

### Productive institutions and individuals

As shown in Table 3 , the University of California system has the largest academic output. As the first author, Fiorella et al. have published the four most cited articles. Most of these highly accomplished individuals were affiliated with the United States and European institutions. There are fewer institutions and individuals from developing countries. This difference may be due to the higher investment in scientific research in western developed countries, which results in strong innovation capabilities and a good academic atmosphere in these countries compared with developing countries.

### Analysis of research type

Among the top 100 most cited reports, clinical studies accounted for 86%, followed by review reports (8%), and basic research (3%). Some clinical research reports have provided high-quality data for clinical decision-making. For example, the International Unruptured Intracranial Aneurysm (ISUIA) study and the ISAT, are examples of multi-center and multi-country cooperation and provided an important reference for the establishment of intravascular treatment norms for IAs. The ISUIA showed that if the diameter of the aneurysm exceeds 7 mm, the risk of rupture is increased. 3 7

We noted that there are few basic research reports. So, it is necessary to strengthen the cooperation in basic research. For instance, we should cooperate to explore the mechanism of hemodynamic changes after endovascular treatment of IAs. A deep understanding of pathogenesis may be beneficial to increase the long-term occlusion rate.

### Limitations of this study

In this study, we only analyzed literature reports in English. This may omit contributions made by non-English authors. Besides, we selected the top 100 most cited articles based on the number of citations, this may underestimate some important articles and overestimate those articles that appeared in the list. Furthermore, the results of bibliometric analysis will change with the update of the number of citations. Moreover, the number of citations used as a reference basis for the influence of the article may not be completely accurate. In the future, it may be more accurate if we can combine multiple indicators to comprehensively judge the influence of the article and then analyze the bibliometrics characteristics of the literature.

In conclusion, this study highlights the top 100 most cited reports in endovascular treatment of IAs. The bibliometric analysis provides insight into the development and growth trend in endovascular treatment of IAs. This study can help researchers to better understand the global overview of this disease and provide information about promising areas of research and potential collaborations.
