# Supplementary material for: Big data analysis of endovascular treatment of intracranial aneurysms: a bibliometric analysis of the top 100 most cited articles
Source: Arq Neuropsiquiatr. 2022 Dec 29;80(12):1189–95. doi: 10.1055/s-0042-1758650 (PMC9800165; doi:10.1055/s-0042-1758650)
Supplement: Supplementary file 1 — Supplementary Material [file 10-1055-s-0042-1758650-s210402.pdf]

Supplementary Table S1 The top 100 most cited articles on endovascular treatment of IAs

Title	Year	Journal	First author	Citation	Citation/ years
International Subarachnoid Aneurysm Trial (ISAT) of neurosurgical clipping versus endovascular coiling in 2143 patients with ruptured intracranial aneurysms: a randomized trial	2002	Lancet	Molyneux, A	2254	112.7
Unruptured intracranial aneurysms: natural history, clinical outcome, and risks of surgical and endovascular treatment	2003	Lancet	Wiebers, D	2068	108.84
International subarachnoid aneurysm trial (ISAT) of neurosurgical clipping versus endovascular coiling in 2143 patients with ruptured intracranial aneurysms: a randomized comparison of effects on survival, dependency, seizures, rebleeding, subgroups, and aneurysm occlusion	2005	Lancet	Molyneux, AJ	1453	85.47
Guglielmi detachable coil embolization of acute intracranial aneurysm: Perioperative anatomical and clinical outcome in 403 patients	1997	Journal of Neurosurgery	Vinuela, F	686	27.44
Curative endovascular reconstruction of cerebral aneurysms with the pipeline embolization device: the Buenos Aires experience	2009	Neurosurgery	Lylyk, Pedro	591	45.46
Guglielmi Detachable Coil embolization of cerebral aneurysms: 11 years' experience	2003	Journal of Neurosurgery	Murayama, Y	567	29.84
The Pipeline Embolization Device for the Intracranial treatment of aneurysms trial	2011	American Journal of Neuroradiology	Nelson, P. K.	510	46.36
Risk of recurrent subarachnoid hemorrhage, death, or dependence and standardised mortality ratios after clipping or coiling of an intracranial aneurysm in the International Subarachnoid Aneurysm Trial (ISAT): long-term follow-up	2009	Lancet Neurology	Molyneux, Andrew J	505	38.85
Endovascular treatment of intracranial aneurysms with Flow Diverters: A Meta-Analysis	2013	Stroke	Brinjikji, Waleed	430	47.78
Endovascular occlusion of intracranial aneurysms with electrically detachable coils	1994	American Journal of Neuroradiology	Zubillaga, AF	422	15.07
Efficient pipeline for image-based patient-specific analysis of cerebral aneurysm hemodynamics: Technique and sensitivity	2005	IEEE Transactions on Medical Imaging	Cebral, JR	407	23.94
Remodeling of saccular cerebral artery aneurysm wall is associated with rupture - Histological analysis of 24 unruptured and 42 ruptured cases	2004	Stroke	Frosen, J	404	22.44
Stent-Assisted Coiling of Intracranial Aneurysms Clinical and Angiographic Results in 216 Consecutive Aneurysms	2010	Stroke	Piotin, Michel	403	33.58
Treatment of intracranial aneurysms by functional reconstruction of the parent artery: The Budapest experience with the Pipeline Embolization Device	2010	American Journal of Neuroradiology	Szikora, I	392	32.67
Five-year experience in using coil embolization for ruptured intracranial aneurysms: outcomes and incidence of late rebleeding	1999	Journal of Neurosurgery	Byrne, JV	390	16.96

 © 2022. Academia Brasileira de Neurologia. All rights reserved.

Supplementary Table S1 (*Continued*)

Title	Year	Journal	First author	Citation	Citation/ years
Reconstruction technique for wide neck intracranial aneurysms: Long-term angiographic and clinical results in a series of 56 cases	1997	Journal of Neuroradiology	Moret, J	364	14.56
Complex hemodynamics at the apex of an arterial bifurcation induces vascular remodeling resembling cerebral aneurysm initiation	2007	Stroke	Meng, Hui	342	22.80
Treatment of intracranial aneurysms by embolization with coils - A systematic review	1999	Stroke	Brilstra, EH	334	14.52
Intravascular stent and endovascular coil placement for a ruptured fusiform aneurysm of the basilar artery - Case report and review of the literature	1997	Journal of Neurosurgery	Higashida, RT	333	13.32
Use of detachable balloons for proximal artery-occlusion in the treatment of unclippable cerebral aneurysms	1987	Journal of Neurosurgery	Fox, AJ	332	9.49
Retreatment of ruptured cerebral aneurysms in patients randomized by coiling or clipping in the international subarachnoid aneurysm trial (ISAT)	2007	Stroke	Campi, A	325	21.66
Long-term angiographic follow-up of 169 intracranial berry aneurysms occluded with detachable coils	1999	Radiology	Cognard, C	324	14.09
Endovascular occlusion of wide-necked aneurysms with a new intracranial microstent (neuroform) and detachable coils	2004	Neurosurgery	Benitez, RP	290	16.11
Intracranial aneurysms treated with the Guglielmi detachable coil: midterm clinical results in a consecutive series of 100 patients	1997	Journal of neurosurgery	Malisch, TW	284	11.83
Neuroform stent-assisted coil embolization of wide-neck intracranial aneurysms: Strategies in stent deployment and midterm follow-up	2007	Neurosurgery	Biondi, A	274	18.27
Outcomes of early endovascular versus surgical treatment of ruptured cerebral aneurysms - A prospective randomized study	2000	Stroke	Koivisto, T	273	12.41
Relation between aneurysm volume, packing, and compaction in 124 cerebral aneurysms treated with coils	2004	Radiology	Sluzewski, M	270	15
Treatment of Intracranial Aneurysms Using the Pipeline Flow-Diverter Embolization Device: A Single-Center Experience with Long-Term Follow-Up Results	2012	American journal of neuroradiology	Saatci, I	265	26.50
Natural history of the neck remnant of a cerebral aneurysm treated with the Guglielmi detachable coil system	2000	Journal of neurosurgery	Hayakawa, M	259	11.77
Coiling of Intracranial Aneurysms A Systematic Review on Initial Occlusion and Reopening and Retreatment Rates	2009	Stroke	Ferns, SP	259	19.92
Deliberate basilar or vertebral artery-occlusion in the treatment of intracranial aneurysms – immediate results and long-term outcome in 201 patients	1993	Journal of neurosurgery	STEINBERG, GK	254	8.75

(*Continued*)

 © 2022. Academia Brasileira de Neurologia. All rights reserved.

Supplementary Table S1 (*Continued*)

Title	Year	Journal	First author	Citation	Citation/ years
Pipeline embolization device (PED) for neurovascular reconstruction: initial experience in the treatment of 101 intracranial aneurysms and dissections	2012	Neuroradiology	Fischer, S	252	25.2
Definitive reconstruction of circumferential fusiform intracranial aneurysms with the pipeline embolization device	2008	Neurosurgery	Fiorella, D	246	17.57
Intracranial berry aneurysms: Angiographic and clinical results after endovascular treatment	1998	Radiology	Cognard, C	245	10.21
Usefulness of the neuroform stent for the treatment of cerebral aneurysms: Results at initial (3–6 months) follow-up	2005	Neurosurgery	Fiorella, D	239	14.06
Preliminary experience using the neuroform stent for the treatment of cerebral aneurysms	2004	Neurosurgery	Fiorella, D	239	13.28
Immediate clinical outcome of patients harboring unruptured intracranial aneurysms treated by endovascular approach - Results of the ATENA study	2008	Stroke	Pierot, L	239	17.07
Efficacy and current limitations of intravascular stents for intracranial internal carotid, vertebral, and basilar artery aneurysms	1999	Journal of neurosurgery	Lanzino, G	227	9.87
Flow-Diverter Stent for the Endovascular Treatment of Intracranial Aneurysms A Prospective Study in 29 Patients With 34 Aneurysms	2010	Stroke	Lubicz, B	226	18.83
Balloon catheter technique for dilatation of constricted cerebral arteries after aneurysmal SAH	1984	Acta neurochirurgica	ZUBKOV, YN	221	8.19
Selection of cerebral aneurysms for treatment using Guglielmi detachable coils: The preliminary university of Illinois at chicago experience	1998	Neurosurgery	Debrun, GM	220	9.17
Endovascular coil occlusion of 1811 intracranial aneurysms: Early angiographic and clinical results	2004	Neurosurgery	Henkes, H Fischer, S	215	11.94
The durability of endovascular coiling versus neurosurgical clipping of ruptured cerebral aneurysms: 18 year follow-up of the UK cohort of the International Subarachnoid Aneurysm Trial (ISAT)	2015	Lancet	Molyneux, AJ	211	30.14
Early experience in the treatment of Intra-Cranial Aneurysms by Endovascular Flow Diversion: A Multicentre Prospective Study	2010	PLoS one	Byrne, JV	207	17.25
Stent-assisted coiling of intracranial aneurysms predictors of complications, recanalization, and outcome in 508 Cases	2013	Stroke	Chalouhi, N	201	25.13
Selective endovascular treatment of 71 intracranial aneurysms with platinum coil	1993	Journal of neurosurgery	CASASCO, AE	193	6.66
Self-expanding and balloon-expandable stents in the treatment of carotid aneurysm-an experimental study in a canine model	1994	American journal of neuroradiology	WAKHLOO, AK	192	6.86

 © 2022. Academia Brasileira de Neurologia. All rights reserved.

Supplementary Table S1 (*Continued*)

Title	Year	Journal	First author	Citation	Citation/years
Clinical and angiographic results of endosaccular coiling treatment of giant and very large intracranial aneurysms: A 7-year, single-center experience	1999	Neurosurgery	Gruber, A	191	8.30
Endovascular detachable balloon embolization therapy of cavernous carotid-artery aneurysms-results in 87 cases	1990	Journal of neurosurgery	HIGASHIDA, RT	183	14.08
Treatment of intracranial aneurysms with the Enterprise stent: a multicenter registry	2009	Journal of neurosurgery	Mocco, J	183	14.08
Rates of delayed rebleeding from intracranial aneurysms are low after surgical and endovascular treatment	2006	Stroke	Johnston, SC	183	11.43
Hydrogel-coated coils versus bare platinum coils for the endovascular treatment of intracranial aneurysms (HELPS): a randomized controlled trial	2011	Lancet	White, PM	179	16.27
Results of treatment of intracranial aneurysms by occlusion of carotid artery in neck	1966	Journal of neurosurgery	NISHIOKA, H	178	3.18
Panacea or problem: flow diverters in the treatment of symptomatic large or giant fusiform vertebrobasilar aneurysms	2012	Journal of neurosurgery	Siddiqui, AH	178	17.80
Endovascular and surgical treatment of unruptured cerebral aneurysms: Comparison of risks	2000	Annals of neurology	Johnston, SC	176	8
Embolization of incidental cerebral aneurysms by using the Guglielmi detachable coil system	1999	Journal of neurosurgery	Murayama, Y	174	7.56
Hunterian proximal arterial-occlusion for giant aneurysms of the carotid circulation	1994	Journal of neurosurgery	DRAKE, CG	173	6.18
Buenos aires experience with the neuroform self-expanding stent for the treatment of intracranial aneurysms	2005	Journal of neurosurgery	Lylyk, P	172	10.12
Ruptured intracranial aneurysms: Acute endovascular treatment with electrolytically detachable coils - A prospective randomized study	1999	Radiology	Vanninen, R	172	7.48
Matrix and Bioabsorbable polymeric coils accelerate healing of intracranial aneurysms-Long-term experimental study	2003	Stroke	Murayama, Y	166	8.74
Follow-up angiography of intracranial aneurysms treated with endovascular placement of Guglielmi detachable coils	2002	Neurosurgery	Thornton, J	165	8.25
Surgical and endovascular treatment of unruptured cerebral aneurysms at university hospitals	1999	Neurosurgery	Johnston, SC	163	7.08
A Multicenter study of 705 ruptured intracranial aneurysms treated with Guglielmi detachable coils	2005	American journal of neuroradiology		162	9.53
Use of etomidate, temporary arterial-occlusion, and intraoperative angiography in surgical-treatment of large and giant cerebral aneurysms	1988	Journal of neurosurgery	BATJER, HH	159	4.67

(*Continued*)

 © 2022. Academia Brasileira de Neurologia. All rights reserved.

Supplementary Table S1 (*Continued*)

Title	Year	Journal	First author	Citation	Citation/ years
Intracranial aneurysms: Clinical value of 3D digital subtraction angiography in the therapeutic decision and endovascular treatment	2001	Radiology	Anxionnat, R	158	7.52
Stenting and secondary coiling of intracranial internal carotid artery aneurysm: Technical case report	1998	Neurosurgery	Mericle, RA	157	6.54
Neurosurgical management of cerebral aneurysms following unsuccessful or incomplete endovascular embolization	1995	Journal of neurosurgery	GURIAN, JH	157	5.81
Stent-Assisted Coiling in Acutely Ruptured intracranial Aneurysms: A Qualitative, Systematic Review of the Literature	2011	American journal of neuroradiology	Bodily, KD	157	15.7
Early fatal hemorrhage after endovascular cerebral aneurysm treatment with a flow diverter (SILK-Stent)	2011	Neuroradiology	Turowski, B	157	15.7
Treatment of internal carotid artery aneurysms with a covered stent: Experience in 24 patients with mid-term follow-up results	2004	American journal of neuroradiology	Saatci, I	156	9.17
Endovascular occlusion of intracranial aneurysms with Guglielmi detachable coils: Correlation between coil packing density and coil compaction	2001	Acta neurochirurgica	Kawanabe, Y	155	7.38
Trans-vascular treatment of giant aneurysms of the cavernous carotid and vertebral arteries-functional investigation and embolization	1984	Surgical neurology	BERENSTEIN, A	155	4.08
High-Profile Flow Diverter (Silk) Implantation in the Basilar Artery Efficacy in the Treatment of Aneurysms and the Role of the Perforators	2010	Stroke	Kulcsar, Z	154	12.83
Self-expandable stent-assisted coiling of wide-necked intracranial aneurysms: A single-center experience	2005	American journal of neuroradiology	Akpek, S	154	9.06
Curative cerebrovascular reconstruction with the Pipeline embolization device: the emergence of definitive endovascular therapy for intracranial aneurysms	2009	Journal of neurointerventional surgery	Fiorella, D	153	11.77
Results of a prospective protocol of computed tomographic angiography in place of catheter angiography as the only diagnostic and pretreatment planning study for cerebral aneurysms by a combined neurovascular team	2004	Neurosurgery	Hoh, BL	152	8.44
Thromboembolic events associated with Guglielmi detachable coil embolization of asymptomatic cerebral aneurysms: Evaluation of 66 consecutive cases with use of diffusion-weighted MR imaging	2003	American journal of neuroradiology	Soeda, A	152	8
Endovascular Treatment of Intracranial Unruptured Aneurysms: Systematic Review and Meta-Analysis of the Literature on Safety and Efficacy	2010	Radiology	Naggara, ON	152	12.67
Endovascular treatment of ruptured intracranial aneurysms with detachable coils: Long-term clinical and serial angiographic results	2003	Radiology	Sluzewski, M	151	7.95

 © 2022. Academia Brasileira de Neurologia. All rights reserved.

Supplementary Table S1 (*Continued*)

Title	Year	Journal	First author	Citation	Citation/years
Comparison of balloon angioplasty and papaverine infusion for the treatment of vasospasm following aneurysmal subarachnoid hemorrhage	1998	Journal of neurosurgery	Elliott, JP	150	6.25
A clinical-study of the parameters and effects of temporary arterial-occlusion in the management of intracranial aneurysms	1994	Neurosurgery	SAMSON, D	149	5.32
Coiling of very large or giant cerebral aneurysms: Long-term clinical and serial angiographic results	2003	American journal of neuroradiology	Sluzewski, M	148	7.79
Endovascular management of vertebrobasilar dissecting aneurysms	2003	American journal of neuroradiology	Rabinov, JD	148	7.79
Intracranial aneurysms: midterm outcome of pipeline embolization device-a prospective study in 143 patients with 178 aneurysms	2012	Radiology	Yu, SCH	148	14.8
Safety and efficacy of adjunctive balloon remodeling during endovascular treatment of intracranial aneurysms: a literature review	2008	American journal of neuroradiology	Shapiro, M	147	10.5
Stents for intracranial aneurysms: The beginning of a new endovascular era?	1998	Neurosurgery	Wakhloo, AK	146	6.08
An Original Flow Diversion Device for the Treatment of Intracranial Aneurysms Evaluation in the Rabbit Elastase-Induced Model	2009	Stroke	Sadasivan, C	146	11.23
Intracranial aneurysms: links among inflammation, hemodynamics and vascular remodeling	2006	Neurological research	Hashimoto, T	146	9.13
Thromboembolic events associated with the treatment of cerebral aneurysms with Guglielmi detachable coils	1998	American journal of neuroradiology	Pelz, DM	146	6.08
Comparison of Flow Diversion and Coiling in Large Unruptured Intracranial Saccular Aneurysms	2013	Stroke	Chalouhi, N	146	16.22
Clipping Versus Coiling for Ruptured Intracranial Aneurysms A Systematic Review and Meta-Analysis	2013	Stroke	Li, H	145	16.11
Vascular extracellular matrix remodeling in cerebral aneurysms	1998	Journal of neurosurgery	Bruno, G	144	6
Flow Diversion for Intracranial Aneurysms A Review	2011	Stroke	Durso, PI	143	13
Rupture of intracranial aneurysms during treatment with Guglielmi detachable coils: incidence, outcome, and risk factors	2001	Journal of neurosurgery	Sluzewski, M	141	6.71
Randomized Trial of Clazosentan in Patients With Aneurysmal Subarachnoid Hemorrhage Undergoing Endovascular Coiling	2012	Stroke	Macdonald, RL	141	14.10
Flow-Diverter Silk Stent for the Treatment of Intracranial Aneurysms: 1-year Follow-Up in a Multicenter Study	2012	American journal of neuroradiology	Berge, J	137	13.70
Endovascular treatment of giant and large intracranial aneurysms by using a combination of stent placement and liquid polymer injection	2002	Journal of neurosurgery	Mawad, ME	137	6.85

(*Continued*)

 © 2022. Academia Brasileira de Neurologia. All rights reserved.

Supplementary Table S1 (*Continued*)

Title	Year	Journal	First author	Citation	Citation/ years
Clinical and angiographic outcomes, with treatment data, for patients with cerebral aneurysms treated with Guglielmi detachable coils: A single-center experience	1998	Neurosurgery	Kuether, TA	136	5.67
Flow Diverters at and Beyond the Level of the Circle of Willis for the Treatment of Intracranial Aneurysms	2012	Stroke	Pistocchi, S	135	13.5
Patency of the ophthalmic artery after flow diversion treatment of paraclinoid aneurysms	2012	Journal of neurosurgery	Puffer, RC	135	13.5

 © 2022. Academia Brasileira de Neurologia. All rights reserved.
